# The Effect of the Chemical Character of Ionic Liquids on Biomass Pre-Treatment and Posterior Enzymatic Hydrolysis

**DOI:** 10.3390/molecules24040808

**Published:** 2019-02-23

**Authors:** Joana R. Bernardo, Francisco M. Gírio, Rafał M. Łukasik

**Affiliations:** Unidade de Bioenergia, Laboratório Nacional de Energia e Geologia, I.P., Estrada do Paço do Lumiar 22, 1649-038 Lisboa, Portugal; joana.bernardo@lneg.pt (J.R.B.); francisco.girio@lneg.pt (F.M.G.)

**Keywords:** biomass, valorisation, ionic liquid, crystallinity, enzymatic hydrolysis, pre-treatment

## Abstract

Ionic liquids have been recognised as interesting solvents applicable in efficient lignocellulosic biomass valorisation, especially in biomass fractionation into individual polymeric components or direct hydrolysis of some biomass fractions. Considering the chemical character of ionic liquids, two different approaches paved the way for the fractionation of biomass. The first strategy integrated a pre-treatment, hydrolysis and conversion of biomass through the employment of hydrogen-bond acidic 1-ethyl-3-methyimidazolim hydrogen sulphate ionic liquid. The second strategy relied on the use of a three-step fractionation process with hydrogen-bond basic 1-ethyl-3-methylimidazolium acetate to produce high purity cellulose, hemicellulose and lignin fractions. The proposed approaches were scrutinised for wheat straw and eucalyptus residues. These different biomasses enabled an understanding that enzymatic hydrolysis yields are dependent on the crystallinity of the pre-treated biomass. The use of acetate based ionic liquid allowed crystalline cellulose I to change to cellulose II and consequently enhanced the glucan to glucose yield to 93.1 ± 4.1 mol% and 82.9 ± 1.2 mol% for wheat straw and eucalyptus, respectively. However, for hydrogen sulphate ionic liquid, the same enzymatic hydrolysis yields were 61.6 ± 0.2 mol% for wheat straw and only 7.9 ± 0.3 mol% for eucalyptus residues. These results demonstrate the importance of both ionic liquid character and biomass type for efficient biomass processing.

## 1. Introduction

Lignocellulosic biomass is a renewable, sustainable, abundant, and CO_2_ neutral alternative to fossil feedstock for a portfolio of fuels, chemicals and materials. Composed of crystalline cellulose nanofibrils embedded in an amorphous matrix of cross-linked lignin and hemicelluloses, lignocellulose shows a natural recalcitrance that impedes enzyme and microbial accessibility, resulting in the relatively low digestibility of raw lignocellulosic materials [1]. Thus, an efficient pre-treatment, and consequently a deconstruction of the lignocellulosic biomass, makes these fractions susceptible for more favourable transformation to value-added products [2,3]. However, many pre-treatment methods require harsh conditions, especially temperature and/or pressure that often result in undesired by-products, which decrease the sugar yields and inhibit enzymatic hydrolysis and further bioconversion [2].

In recent years, ionic liquids (ILs) have gained increasing interest for biomass processing due to their capacity to dissolve lignocellulosic biomass by an effective disruption of the complex network of noncovalent interactions between carbohydrates and lignin [4,5,6]. A main function of IL in lignocellulosic biomass pre-treatment is the modification the fibrillary structure of cell walls in order to: (i) decrease cellulose crystallinity, (ii) increase cellulose surface accessibility by the removal of lignin and/or hemicellulose, and (iii) promotion of a swelling effect of the biomass [7]. Imidazolium-based ILs are among the most extensively studied ILs and have demonstrated that either a cation or an anion is of considerable importance in biomass processing [8,9]. Swatloski et al. showed that a high concentration of chloride anions is effective in breaking down the hydrogen-bond network of cellulose [10]. A similar effect was reported for the acetate ([OAc]) anion, which was demonstrated to be efficient in the dissolution of lignocellulosic biomass [11]. It was reported that a key reason for this was the high hydrogen bond acceptor capacity (β) of the [OAc] anion (β = 1.201) in comparison to previously mentioned chloride anion (β = 0.83) [12]. Due to this, 1-ethyl-3-methylimidazolium acetate IL is confirmed to be one of the best and is one of the most commonly used [13] ILs, able to dissolve a large variety of lignocellulosic biomass and to fractionate it into cellulose- and hemicellulose-rich fractions, as well as to produce high pure lignin [9,14,15].

An alternative approach to biomass processing with ILs is the employment of ILs as both solvent and catalyst. In these processes, ILs hydrolyse mainly polysaccharide fractions without the presence of other catalysts [5,16,17]. Therefore, ILs with an acidic character have demonstrated an ability to selectively hydrolyse hemicellulose [18,19], both cellulose and hemicellulose [20], or lignin [21]. Some of the most commonly used ILs in such approaches are those based on hydrogen sulphate ([HSO_4_]). They are able to catalyse a selective hemicellulose hydrolysis [17,18,19]. Furthermore, [HSO_4_]-based ILs have been increasingly used because of their acidic properties and due to their low cost when compared to other ILs [22]. The use of acidic IL, e.g., 1-butyl-3-methylimidazolium hydrogen sulphate, [bmim][HSO_4_], allowed achievement of up to 90% fermentable glucose after enzymatic saccharification of the cellulose-rich *Miscanthus* pulp [18]. The same IL was also reported as being able to hydrolyse and to convert the hemicellulose fraction of wheat straw with no additional catalyst used [17]. The pre-treatment with this IL produced a liquor enriched in hemicellulosic sugars, furans and organic acids, and a solid fraction constituted mainly of cellulose and lignin. Furthermore, water was confirmed to have an influence on the equilibrium of the hemicellulose hydrolysis. The increase of the water content close to 10% (*w*/*w*) in the reaction system disfavoured xylose dehydration, and thus allowed the production of hemicellulose-derived monosaccharides to increase significantly [19].

Building on previous works about the processing of biomass with hydrogen-bond basic ([emim][OAc]) [11] and hydrogen-bond acidic ([emim][HSO_4_]) [17,19] ILs, this work aimed to demonstrate the importance of biomass type on the efficiency of the biomass pre-treatment, as well as on the efficiency of subsequent enzymatic hydrolysis. This was examined using two very distinct types of biomasses, namely, herbaceous (wheat straw) and hardwood *Eucalyptus globulus*, allowing the elucidation of changes observed in the chemical structure of the biomass, cellulose crystallinity and consequently the effects of these on the cellulose-rich pulp hydrolysability.

## 2. Results and Discussion

### 2.1. Hydrogen-Bond Acidic IL

#### 2.1.1. Biomass Pre-Treatment with [emim][HSO_4_]

The first methodology used focused on the biomass pre-treatment with [emim][HSO_4_]. As stated above, this approach allowed the integration of biomass pre-treatment, hydrolysis and conversion in a single-step process. The acidic character of the [HSO_4_] anion of IL, promoted a selective hydrolysis of the hemicellulose fraction, and the resulting products (mainly pentoses and furfural) were kept in the liquid phase. Both biomasses, wheat straw and eucalyptus residues, were subject to processing with [emim][HSO_4_] IL at 140 °C for 90 min at varied [emim][HSO_4_]/H_2_O mass ratio and with fixed 10 wt.% of dry biomass in the reaction mixture.

**Figure 1:**
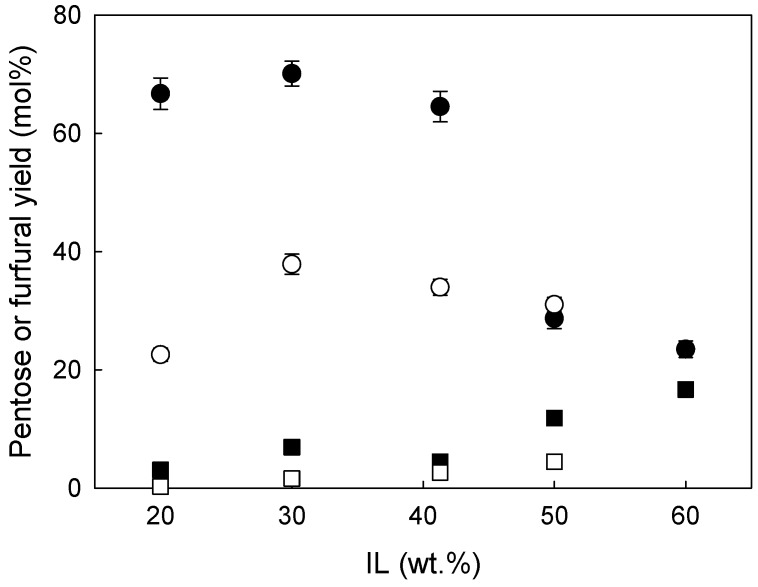
The yields of (●○) pentose (sum of arabinose and xylose) or (■□) furfural as a function of ionic liquid (IL) concentration (wt.%) obtained from pre-treatment of wheat straw (filled symbols) and eucalyptus residues (open symbols) performed at 140 °C for 90 min.

The pentose and furfural yields obtained in these trials are depicted in Figure 1.

**Figure 2:**
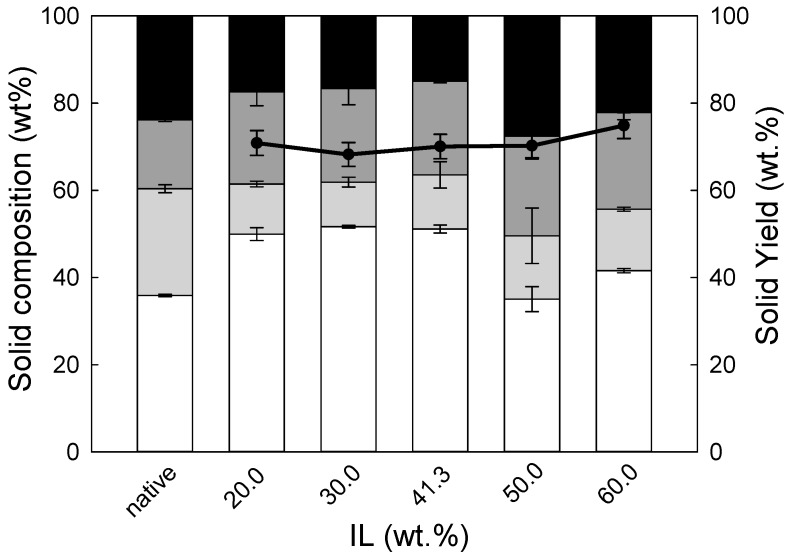
The solid phase composition (white bar–cellulose; light grey bar–hemicellulose; dark grey bar–Klason lignin; black bar–others) of wheat straw pre-treatment at 140 °C and 90 min with [emim][HSO_4_] at various % IL. The solid line represents the solid yield (wt.%) of pre-treated solids recovered after pre-treatment.

As can be seen, at IL concentration of 30 wt.%, the pentose yields peaked for both biomasses. For eucalyptus residues or wheat straw, a further increase in the IL concentration was demonstrated to have a negative effect on the pentose yield as it was counterbalanced by predominant production of furfural. These results are not surprising since a high performance of acidic ILs towards furfural is often reported in the literature [23,24,25]. One of the reasons for this is that pentose conversion to furfural is a dehydration reaction; therefore, a higher concentration of IL, or in other words a lower concentration of water, may disturb the equilibrium existing in the system promoting the dehydration of pentoses towards furfural production. On the other hand, the presence of water in the system allowed protection of pentoses from dehydration, and consequently, it was possible to obtain a high pentose yield for IL concentration below 30 wt.%. Although this observation is valid for either wheat straw or eucalyptus residues, the yield of hemicellulose hydrolysis, as well as pentose and furfural yields, seems to be strongly dependent on the nature of the biomass. As can be seen in Figure 1, in the best conditions, i.e., 30 wt.% IL, pre-treatment of the eucalyptus residues with [emim][HSO_4_] allowed achievement of only 37.9 ± 1.7 mol% pentoses, while for wheat straw the maximum pentose concentration was almost double, i.e., 70.1 ± 0.5 mol%. This difference is also reflected in the composition of pre-treated solids and the solid yields. The latter was very high for eucalyptus residues and varied between 81 and 85 wt.%, while for pre-treated wheat straw it oscillated between 68 and 75 wt.%. For both biomasses, the lowest solid yields were observed for solids obtained following pre-treatment with an IL concentration of 30 wt.%. This is a direct consequence of the most pronounced hydrolysis of hemicellulose. Further increase in the IL concentration increased the solid yield for wheat straw up to 74.9 ± 3.0 wt.%. This unexpected increase of the solid yield with an increase in the pre-treatment intensity might be justified by the formation of pseudo-lignins, also called humins [26]. As pseudo-lignins are quantified gravimetrically [27], they contribute to an increase in the lignin content detected, which is clearly visible in Figure 2. For two the highest IL concentrations tested, the lignins recovered exceeded 100 wt.% of the lignin present in the native biomass. This, in turn may confirm this hypothesis.

**Figure 3:**
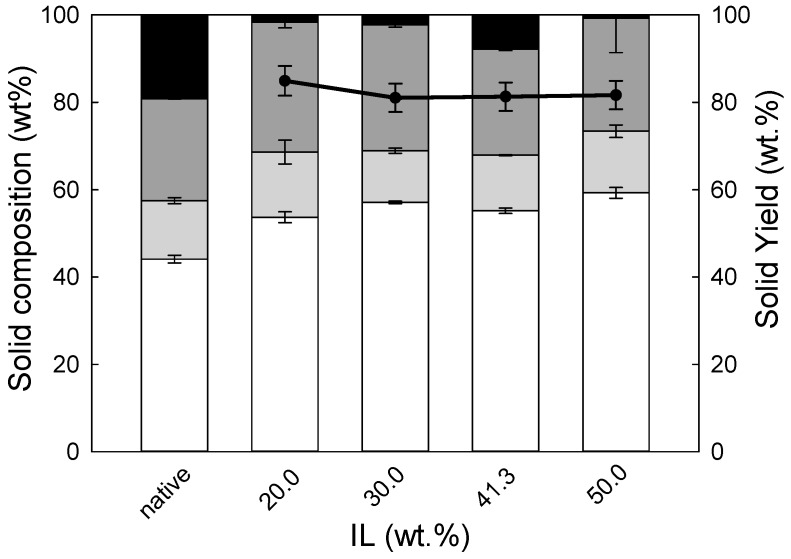
The solid phase composition (white bar–cellulose; light grey bar–hemicellulose; dark grey bar–Klason lignin; black bar–others) of eucalyptus residues pre-treatment at 140 °C and 90 min with [emim][HSO_4_] at various % IL. The solid line represents the solid yield (wt.%) of pre-treated solids recovered after pre-treatment.

Similar effects were not observed for eucalyptus residues, for which either solid yield appeared to be constant or the lignin content in the solids pre-treated at various IL concentrations did not change, as demonstrated in Figure 3.

The aforementioned analysis of the hemicellulose hydrolysis also reflects the macromolecular composition of the pre-treated leftovers of both biomasses. It is clear that the IL pre-treatment induced the reduction of the hemicellulose fraction in comparison to native biomasses, as xylan was found in amounts not exceeding 15 wt.% of the pre-treated biomasses. Consequently, the major components of the solids produced were glucan, followed by lignin, as already discussed. This indicates that contrary to a great performance by aqueous [emim][HSO_4_] solution in processing hemicellulose, a cellulose hydrolysis in this catalytic system seems to be very inefficient. For IL concentrations up to the studied 41.3 wt.%, the cellulose yield was kept constant, but in case of wheat straw, for higher IL concentrations (>50 wt.%), the cellulose content started to diminish. This demonstrates that, only under these conditions, the cellulose fraction of wheat straw was also susceptible to hydrolysis, and for pre-treatment with IL concentration of 60 wt.% as much as 20 wt.% of cellulose, in comparison to native biomass, was removed. In the case of eucalyptus residues, in the range of IL concentrations studied, 20 wt.% of IL already allowed the removal of about 1/3 of hemicellulose and further increases in the IL concentration in the reaction mixture resulted in no further significant changes in the composition of the pre-treated solids, as can be seen in Figure 3. These results confirm again that although ILs are capable of processing various types of herbaceous and woody biomass as a single feedstock, the conditions for efficient pre-treatment are dependent upon the biomass type, with softwoods or hardwoods recognised as the most difficult to process when compared to herbaceous biomass [6]. Indeed, Xu et al. found that the eucalyptus residues treatment catalysed by 0.5% (v⋅v^−1^) [bmim][HSO_4_] yielded only ≈ 25 mol% of pentose at 190 °C [28]. Other reports in the literature depict the use of the acidic ILs in the pre-treatment of biomass as well. For example, Li et al. demonstrated acidic IL to be an efficient system for the hydrolysis of lignocellulosic materials. They obtained total reducing sugars (TRS) yields of 23% and 15% from corn stalk after only 5 min of the reaction at 100 °C in [bmim][HSO_4_] and [C_4_SO_3_Hmim][HSO_4_], respectively. On the other hand, longer reaction times provided lower TRS yields, suggesting that these strongly acidic ILs resulted in the promotion of more advanced degradation of polysaccharide fraction at the pre-treatment step [20]. Other work showed that in case of *Miscanthus*, pre-treatment at a higher temperature (120 °C) and for 4 h with [bmim][HSO_4_] and 20 vol.% (17 wt.%) of H_2_O content, close to 16% of hemicellulose sugar monomers were obtained [18]. In a different study, Carvalho et al. showed that the same IL with 9.22 wt.% H_2_O content in the pre-treatment of wheat straw (125 °C and 82.1 min) allowed a 40.1 mol% pentose yield to be obtained [19]. Thus, comparing the data in the literature to the data presented in this work, it can be stated that a higher yield of pentoses was selectively achieved without excessive amounts of IL being present in the system.

#### 2.1.2. Enzymatic Hydrolysis of Pre-Treated Solids

**Figure 4:**
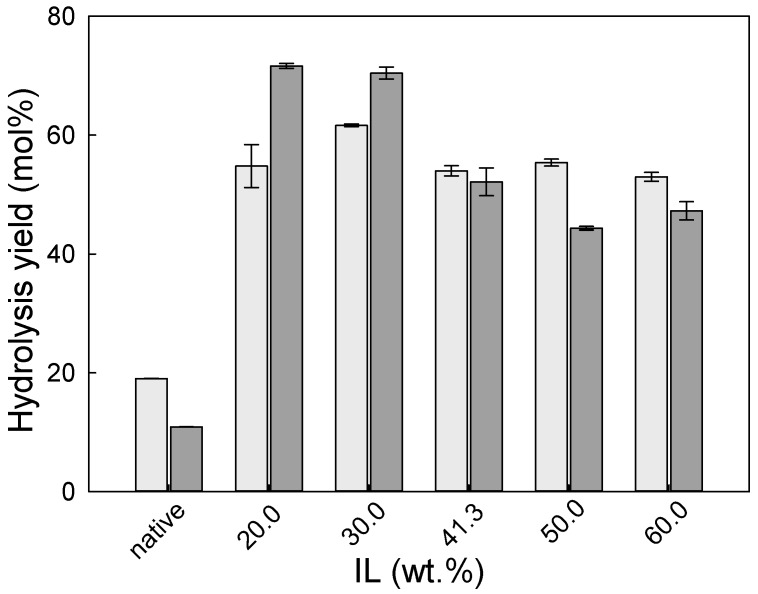
The enzymatic hydrolysis yield (glucan to glucose–light grey bar; and xylan to xylose–dark grey bar) of wheat straw pre-treated solids as a function of IL concentration used in the pre-treatment. The enzymatic hydrolysis yield for native wheat straw is presented for comparison.

The efficiency of pre-treatment of wheat straw and eucalyptus residues by [emim][HSO_4_] was evaluated by enzymatic hydrolysis. The enzymatic hydrolysis yield of pre-treated wheat straw is presented in Figure 4.

As can be observed in Figure 4, the pre-treatment of wheat straw with [emim][HSO_4_] allowed the enzymatic hydrolysis of cellulose to glucose to increase by 3-fold in comparison to native biomass. Interestingly, the enzymatic hydrolysis yield of cellulose (light grey bars) changed only slightly with the IL concentration used in pre-treatment and varied from 53.0 ± 0.7 mol.% to 61.6 ± 0.2 mol% for 60 and 30 wt.% of IL, respectively. On the other hand, the xylan hydrolysis yield decrease was more pronounced, with an increase of IL concentration from 71.7 ± 0.4 mol% to 47.3 ± 1.5 mol% for 20 and 60 wt.% of IL concentration, respectively. The reason for this might be that with an increase in the reaction intensity, more hemicellulose was extracted from the biomass, and consequently less hemicellulose accessible for enzymatic attack was present in the pre-treated solid. In addition, the formation of pseudo-humins may interfere with the accessibility of enzymes to hemicellulose, and lignin presence affects an enzymatic hydrolysis because of unproductive and irreversible cellulase enzyme adsorption on lignin [29].

**Figure 5:**
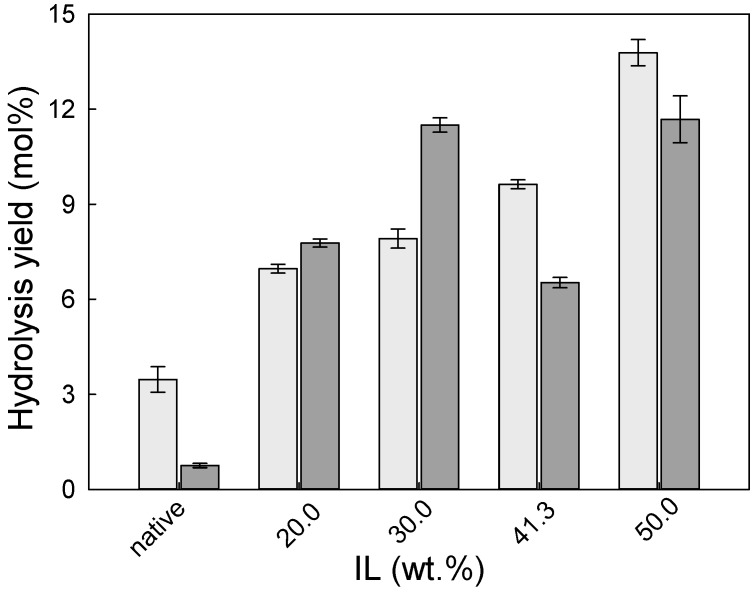
The enzymatic hydrolysis yield (glucan to glucose–light grey bar; and xylan to xylose–dark grey bar) of eucalyptus residues pre-treated solids as a function of IL concentration used in the pre-treatment. The enzymatic hydrolysis yield for native eucalyptus residues is presented for comparison.

Analogous to wheat straw, pre-treatment of eucalyptus residues with [emim][HSO_4_] improved the enzymatic hydrolysability in comparison to native biomass as can be seen in Figure 5.

Furthermore, for eucalyptus residues pre-treated solids, an increase of IL concentration had a generally positive effect on the yield of enzymatic hydrolysis. For example, an enhancement of glucan to glucose yield was found with an increase of IL concentration; however, the hydrolysis yields detected were very low and did not exceed 14 mol%, i.e., 4.5-fold lower than observed for wheat straw. In previous work, Xu et al. verified that the cellulose-rich solids from eucalyptus residues after [bmim][HSO_4_]-catalysed hydrothermal microwave pre-treatment, allowed the achievement of greater glucose conversion yield (89.2%). However, this was only possible when temperatures as high as 200 °C were used in pre-treatment and enzymatic hydrolysis was performed at 2 *w*/*v*% of substrate concentrations and 20 FPU/g substrate after 72 h [28]. Brand et al. demonstrated that the pre-treatment time has a significant effect on the enzymatic saccharification [18]. The pre-treatment of *Miscanthus* with [bmim][HSO_4_] at 120 °C for 8 h resulted in a solid which produced ~80% glucose and ~30% hemicellulose release. It is noteworthy that enzymatic saccharification was performed according to very favourable NREL conditions for enzymatic hydrolysis [30].

These very different results for wheat straw and eucalyptus residues drove us to employ the approach with hydrogen-bond basic IL, namely [emim][OAc].

### 2.2. Hydrogen-Bond Basic IL

#### 2.2.1. Biomass Pre-Treatment with [emim][OAc]

**Figure 6:**
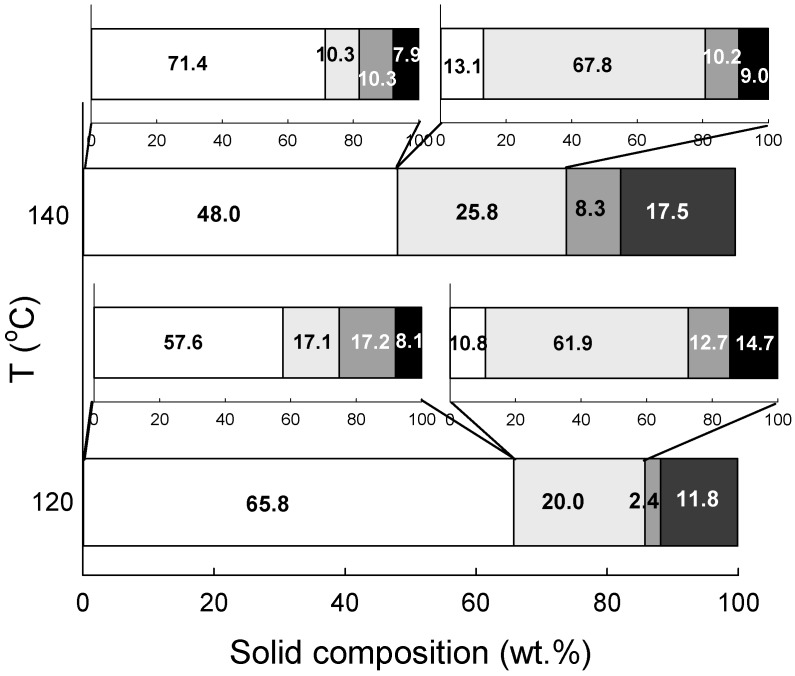
The cellulose- (white bar), hemicellulose- (light grey bar), and Klason lignin-rich (dark grey bar) fractions obtained from wheat straw pre-treated with [emim][OAc] at 120 °C and 140 °C and 2 h. Inserts present the composition of cellulose- and hemicellulose-rich fractions (the same colours were used to differentiate each individual component). Numbers presented in the figure indicate the composition (expressed in wt.%). For exact values, refer to Table S1 in the Electronic Supplementary Information (ESI). Black bars correspond to other non-identified components and were calculated as the difference between biomass used for process or characterised fraction and the cellulose, hemicellulose and lignin determined.

**Figure 7:**
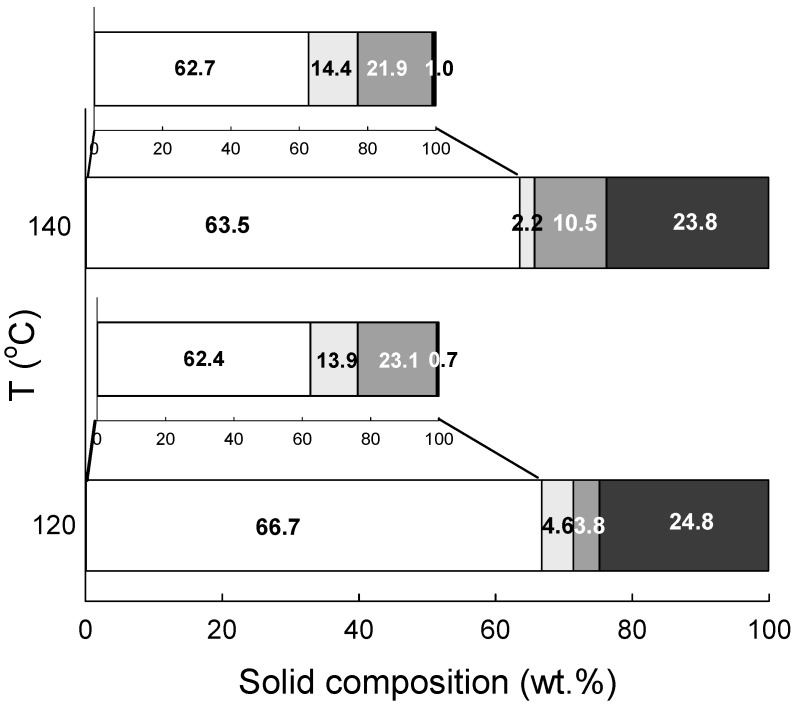
The cellulose- (white bar), hemicellulose- (light grey bar), and Klason lignin-rich (dark grey bar) fractions obtained from eucalyptus residues pre-treated with [emim][OAc] at 120 °C and 140 °C and 2 h. Inserts present the composition of cellulose- and hemicellulose-rich fractions (the same colours were used to differentiate each individual component). Numbers presented in the figure indicate the composition (expressed in wt.%). For exact values, refer to Table S2 in the ESI. Black bars correspond to other non-identified components and were calculated as the difference between biomass used for process or characterised fraction and cellulose, hemicellulose and lignin.

The biomass pre-treatment with hydrogen-bond basic [emim][OAc] IL allowed biomass dissolution and fractionation into cellulose-, hemicellulose- and lignin-rich fractions. For this purpose, pre-treatment of wheat straw or eucalyptus residues in [emim][OAc] at 120 and 140 °C, for 2 h at a biomass/IL ratio of 1/20 (*w*/*w*) was examined on the basis of the methodology presented in the literature [11]. The composition of the obtained solids is given in Figure 6 and Figure 7.

The results obtained demonstrate that temperature has an effect on the selectivity of the fractionation because, although a significantly lower amount of cellulose-rich fraction was obtained at 140 °C in comparison to 120 °C (48.0 wt.% vs. 65.8 wt.% for 140 and 120 °C, respectively), it contained more cellulose, i.e., 57.6 ± 0.3 wt.% vs. 71.4 ± 0.6 wt.% for 120 and 140 °C, respectively. However, as can be seen in Figure 6, a major reason for this was insufficient fractionation of hemicellulose and lignin because the cellulose-rich sample obtained at 120 °C still contained 17.1 ± 3.4 and 17.2 ± 1.0 wt.% of hemicellulose and lignin, respectively. The same sample obtained at 140 °C had much lower hemicellulose and lignin content with only 10.3 ± 1.8 wt.% of each. These relevant differences in more selective fractionation of biomass achieved at higher temperatures also found confirmation in the hemicellulose-rich sample. Contrary to the cellulose-rich sample, the hemicellulose-rich fraction obtained at 140 °C contained more solid, i.e., 25.8 wt.% in comparison to 20.0 wt.% for 120 °C, and this solid was enriched in hemicellulose as it encompassed 67.8 ± 1.6 wt.% of hemicellulose, while at 120 °C it was only 61.9 ± 1.5 wt.%. Consequently, it was predominantly counterbalanced by a difference in lignin and other component contents found in both hemicellulose-rich solids. Interestingly, the lignin-rich fraction obtained at 120 °C was negligible (2.4 wt.%), while that produced at 140 °C was more than three times higher and was equal to 8.3 wt.%. This again clearly indicates that a higher temperature was more effective in the fractionation of wheat straw and allowed the production of cellulose-, hemicellulose- and lignin-rich fractions characterised by higher purity.

Labbé et al. also concluded that at high temperatures, [emim][OAc] is able to cleave the acetyl groups covalently attached, mostly to the hemicellulose component of yellow poplar [31]. Therefore, at a higher temperature this IL can effectively disrupt the carbohydrate–lignin linkages favouring hemicellulose release.

As demonstrated in Figure 7, eucalyptus residues processing with [emim][OAc] at different temperature has almost no effect on the selectivity of biomass fractionation. An increase of temperature by 20 °C from 120 to 140 °C, similar to wheat straw, reduced the amount of cellulose-rich sample recovered from 66.7 wt.% to 63.5 wt.% and enhanced its purity by less than 1 wt.%. Although the trends are the same as those observed for wheat straw, the changes are negligible when compared to those presented in Figure 6. Hemicellulose-rich fractions were recovered in very small quantities, which made their characterisation impossible. Consequently, it can be concluded that although biomass fractionation to cellulose-, hemicellulose- and lignin-rich fraction occurred the eucalyptus residues make the process with hydrogen-bond basic IL less efficient than is the case for herbaceous biomass and others presented in the literature [9,11,32,33].

#### 2.2.2. Enzymatic Hydrolysis of Pre-Treated Solids

**Figure 8:**
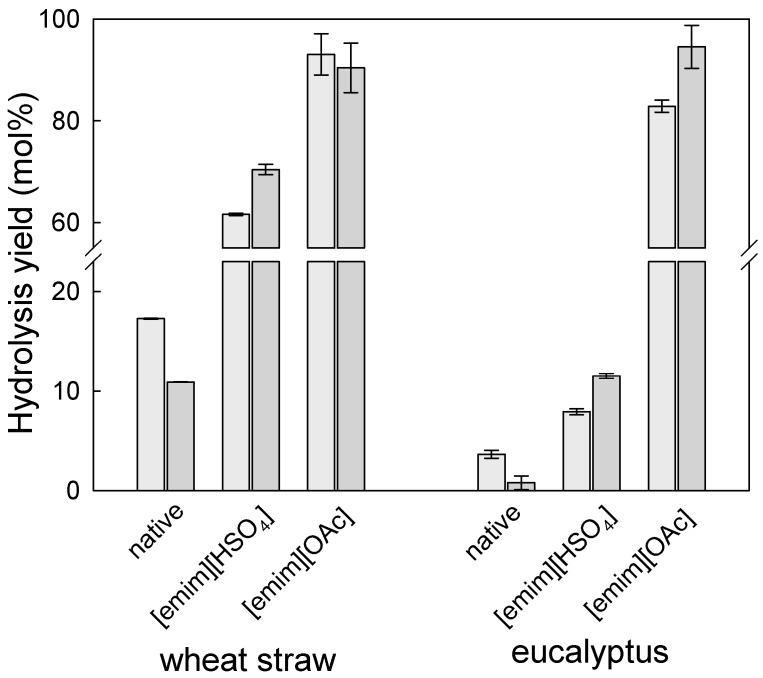
The enzymatic hydrolysis yield (glucan to glucose–light grey bar; and xylan to xylose–dark grey bar) of pre-treated wheat straw and eucalyptus residues solids produced in processes at 140 °C with aqueous [emim][HSO_4_] (30 wt.%, 90 min) and [emim][OAc] (2 h). The enzymatic hydrolysis yield for native biomasses is presented for comparison.

Regardless the efficiency of both biomass fractionation processes, cellulose-rich samples were subject to the enzymatic hydrolysis according to the method presented in the experimental section. The obtained results are presented in Figure 8 and are compared to those achieved for hydrogen-bond acidic IL.

The results obtained demonstrate clearly that pre-treatment of either wheat straw or eucalyptus residues with [emim][OAc] dramatically enhanced the enzymatic hydrolysis yields. For example, for the cellulose-rich sample of wheat straw obtained from pre-treatment with [emim][OAc], the glucan to glucose yield was as high as 93.1 ± 4.1 mol%, while for the same biomass pre-treated with [emim][HSO_4_] a maximum hydrolysis yield of 61.6 ± 0.2 mol% was achieved. Although this 50% enhancement is remarkable, the same process for eucalyptus residues demonstrated an even more astonishing improvement of glucan to glucose hydrolysis yield. As was shown above, the pre-treated solids obtained after reaction with [emim][HSO_4_] allowed achievement of a very modest enzymatic hydrolysis yield of 7.9 ± 0.3 mol%, but the enzymatic hydrolysis yield of cellulose-rich solid obtained from the pre-treatment of eucalyptus residues with [emim][OAc] was as high as 82.9 ± 1.2 mol%. In terms of potential valorisation of cellulose present in the native biomass, a switch from [emim][HSO_4_] to [emim][OAc] was also demonstrated to be a better choice for both biomasses. The yield of glucose released after both steps (pre-treatment with [emim][OAc] and posterior enzymatic hydrolysis) was as high as 91.4 mol% and 74.9 mol% for wheat straw and eucalyptus residues, respectively. The same yield for both biomasses pre-treated with [emim][HSO_4_] with 30 wt.% of IL was only 62.3 mol% and 7.9 mol% for wheat straw and eucalyptus, respectively.

These results indicate that a change of the IL used, from hydrogen-bond acidic to hydrogen-bond basic IL, promoted a significant change in the pre-treated solids, as the enzymatic hydrolysis yield increased by more than 1000%. The results in the literature also confirm similar, although not such pronounced, differences. For example, Bian et al. studied the effect of IL pre-treatment on enzymatic hydrolysis of cellulose as a function of chemical and physical structure changes [34]. In a case of cellulose isolated from sugarcane bagasse subjected to IL ([emim][OAc]), dissolution at a mild temperature (90 °C) followed by a solid regeneration in water, resulted in an increase in glucose content from 80.0–83.3 wt.% to 91.6–92.8 wt.%, a decrease in the degree of polymerisation from 974–1039 units to 511–521 units, a transformation from cellulose I to cellulose II, and an increase of surface area during the pre-treatment. Such cellulose was subsequently hydrolysed by commercial cellulases with 2 *w*/*v*% cellulose substrate and enzyme loadings of 35 FPU/g (cellulase) and 40 CbU (β-glucosidase) in relation to the dry weight of cellulose substrates, and allowed achievement of a high glucose conversion yield of 95.2 mol%. These results suggest that pre-treatment led to an effective disruption of cellulose favouring enzyme hydrolysis. Torr et al. also observed an improvement of the glucan to glucose yield after saccharification for 72 h performed at biomass loading of 1.5% (*w*/*v*) and Celluclast 1.5 L and Novozymes 188 with 40 FPUs/g glucan and β-glucosidades of 50 IU/g glucan, from 5 mol% in the untreated pine wood to 84 mol% in wood pre-treated with [emim][OAc]. The analysis of the substrates revealed that the most important change brought by the pre-treatment was an increase in the accessible surface area. In this case, the delignification was not observed, and loss of cellulose crystallinity only occurred for the highest intensity pre-treatments [35]. Therefore, to verify this, changes in the morphology of pre-treated solid materials were studied using X-ray diffraction.

### 2.3. Morphological Analysis of Pre-Treated Solids

**Figure 9:**
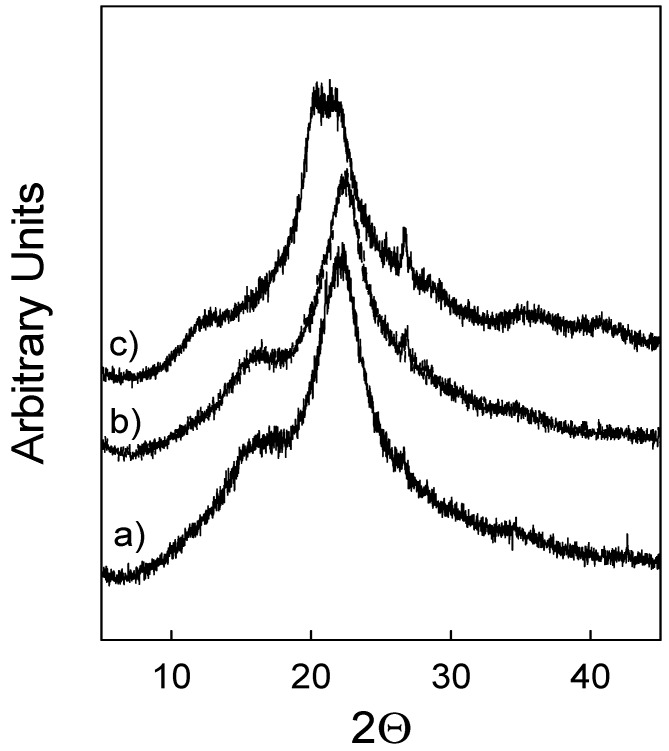
Powder XRD patterns of: (**a**) native wheat straw, (**b**) pre-treated with [emim][HSO_4_], and (**c**) pre-treated with [emim][OAc] IL.

**Figure 10:**
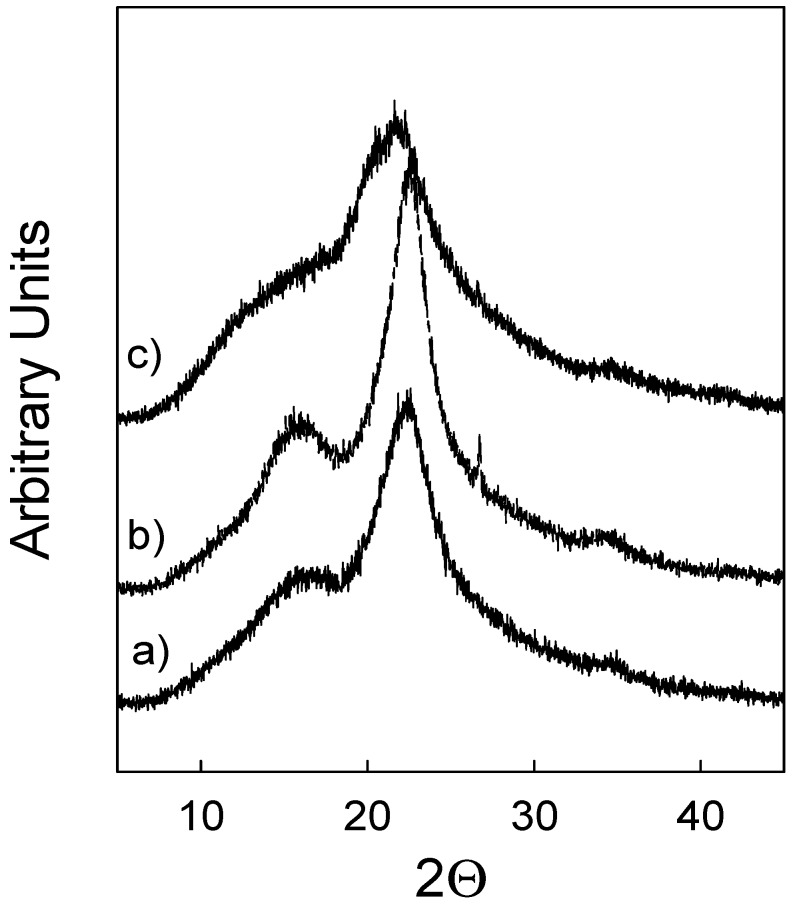
Powder XRD patterns of: (**a**) native eucalyptus residues, (**b**) pre-treated with [emim][HSO_4_] and (**c**) pre-treated with [emim][OAc] IL.

Crystalline cellulose is the most organised form of cellulose in the biomass [36]. Also, crystallinity of cellulose has been reported as one of the most relevant factors influencing the efficiency of enzymatic hydrolysis [37]. X-ray diffraction permits measurement of the crystallinity of the material as a whole, because it demonstrates either crystalline (organised) or disordered components of the biomass, namely, amorphous cellulose, hemicellulose and lignin [38]. ILs have been shown to affect cellulose crystallinity during pre-treatment and consequently enhancing the enzymatic hydrolysis [39]. Therefore, the effect of different pre-treatment approaches on crystallinity, which could justify the efficiency of enzymatic hydrolysis, was also tested in this work. The results obtained for wheat straw and eucalyptus residues are depicted in Figure 9 and Figure 10, respectively.

Figure 9a and Figure 10a show the diffraction patterns of untreated wheat straw and eucalyptus residues. The main signal can be observed at 22.3° for wheat straw and at 22.5° for eucalyptus residue samples. This signal indicates the distance between hydrogen-bonded sheets in cellulose I and corresponds to the (200) lattice plane. For both biomasses, the second main band observed is a broad signal registered at 2θ = 16.7° and corresponds to overlapping signals of (101) and (10-1) planes. The “valley” at 18.1° is associated with an amorphous region in the biomass and includes disordered cellulose, hemicellulose and lignin. The third, barely noticeable, signal at 34.5° corresponds to one-quarter of the length of one cellobiose unit and arises from ordering along the fibre direction.

The XRD patterns of wheat straw and eucalyptus residues pre-treated with [emim][HSO_4_] indicated in Figure 9b and Figure 10b as diffractograms, respectively, mirror the diffractograms observed for native biomasses (Figure 9a and Figure 10a). This confirms that hydrogen-bond acidic ILs, such as [emim][HSO_4_] tested in this work, do not induce any qualitative changes in the pre-treated samples. The same cannot be said about the cellulose-rich solids produced in the [emim][OAc] pre-treatment. The most dominant change is a disappearance of signal at 22.5°, and the presence of a broad asymmetric signal consisting of a doublet at 20° and 21.7°, as demonstrated in Figure 9c. Similarly, the broad signal at 16° disappeared and was substituted with a new signal that emerged at 12.1°, as can be seen in the same figure. These changes are characteristic of a transformation of cellulose I to cellulose II and they are the most visible for wheat straw pre-treated samples. For the cellulose-rich sample of eucalyptus residues, similar changes in the diffractogram are visible although they are less notable. For example, as shown in Figure 10c, a broad signal at 20–22° can be found. Additionally, a signal at 16° became very wide and was transformed into the arm of the main signal. These changes in signals confirm the alteration in the cellulose organisation similar to what was observed for wheat straw. At the same time, as these signals are still not complete defined, contrary to what was observed for the wheat straw cellulose-rich sample, it may indicate that in case of eucalyptus residues, the transformation of crystalline cellulose is less effective and may require longer pre-treatment time. Regardless of the reasons, the observed changes in the XRD patterns justify the fact that even partial alteration of biomass crystallinity is sufficient to promote more efficient enzymatic hydrolysis, as demonstrated in this work. Similar results were presented in the literature, where it was confirmed that cellulose II is more readily digested than cellulose I. It has been argued that the van der Waals interactions between hydrogen-bonded sheets in cellulose I are stronger than in cellulose II and that they act as the main factor in resisting cellulose hydrolysis [40]. Li et al. compared the pre-treatment of switchgrass with [emim][OAc] and with a 1.2% (*w*/*w*) dilute sulphuric acid [41]. For both untreated and dilute acid pre-treated switchgrass samples, little or no change in cellulose crystallinity was observed, but for the sample obtained after pre-treatment with [emim][OAc] the crystallinity was altered significantly, with a structural transformation from cellulose I to cellulose II observed. This, in turn, promoted better performance of enzymatic hydrolysis.

## 3. Materials and Methods

### 3.1. Materials

The wheat straw sample was delivered by ECN (Energy Research Centre of the Netherlands,), from Petten, the Netherlands. The eucalyptus residues were kindly provided by The Navigator Company from their paper mill in Cacia, Portugal. The wheat straw and eucalyptus residues moisture content was found to be 9.8 and 8.4% (*w*/*w*), respectively and was determined using an AMB-50 moisture analyser.

Both feedstocks were ground with a knife mill IKA^®^ WERKE, MF 10 basic (Staufen, Germany) to particles smaller than 0.5 mm, homogenised in a defined lot, and stored in plastic containers at room temperature prior to further use.

The [emim][HSO_4_] IL (99 wt.% of purity) was purchased from Iolitec GmBH—Heilbronn, Germany and was used in reactions without any previous purification. The water content in IL was determined by a volumetric Karl-Fischer titration and was 3796 ppm. The [emim][OAc] with stated purity >95% was purchased from Iolitec GmbH—Heilbronn, Germany. Prior to use in the pre-treatment, this IL was subject to drying under vacuum (0.1 Pa) at room temperature for at least 24 h. The water content in this IL was 2800 ppm, determined by a volumetric Karl-Fischer titration as for the other IL.

In pre-treatment experiments, the following reagents were used: 0.1 M and 3% (*w*/*w*) NaOH aqueous solutions prepared from NaOH pellets (99% purity) supplied by Eka chemicals/Akzonobel–Bohus, Sweden. The aqueous solutions of 1 M and 4 M HCl were prepared from fuming HCl 37% (*w*/*w*) with a purity grade for analysis (Merck—Darmstadt, Germany). Ethanol 96% (*v*/*v*) and acetonitrile of HPLC-gradient purity for analysis (Carlo Erba Group—Arese, Italy) and acetone (98% purity) were supplied by Valente & Ribeiro, Lda.—Belas, Portugal. For the preparation of NaOH and HCl solutions, distilled water (17 M Ω cm^−1^) and ultrapure water (18.2 MΩ cm^−1^) both produced by the PURELAB Classic of Elga system were used.

For filtration, paper and glass microfibre filters (Whatman GE Healthcare Bio-Sciences Corp.—Piscataway, NJ, USA) and nylon filters, 0.45 lm HNPW (Merck Millipore—Billerica, MA, USA) were used.

Glucose (≥98 wt.%, Merck—Darmstadt, Germany), xylose (≥98 wt.%, Merck, Germany), arabinose (≥98 wt.%, Merck, Germany), furfural (furan-2-carbaldehyde) (99 wt.%, Sigma-Aldrich—Steinheim, Germany), 5-hydroxymethylfurfural (5-(hydroxymethyl)-2-furaldehyde) (99 wt.%, Sigma-Aldrich, Germany) and acetic acid (glacial, 99.8 wt.% Merck—Darmstadt, Germany) were used for the qualitative and quantitative HPLC analyses of the obtained liquids and solids. Sulphuric acid (96 wt.%, Panreac—Castellar del Vallès, Spain) was used to prepare mobile phase for HPLC analyses (5 mM sulphuric acid).

For the enzymatic hydrolysis assays, 0.1 M sodium citrate buffer (pH 4.8) prepared from citric acid monohydrate (99.7% purity) and tris-sodium citrate (>99% purity) both from VWR International Ltd.—Leicester, England and a 2 wt.% sodium azide solution were used. Celli^®^CTec2 enzyme solution kindly provided by Novozymes A/S Europe—Bagsvaerd, Denmark was employed in the enzymatic reaction.

### 3.2. Biomass and Pre-Treated Solid Characterisation

Both biomasses and pre-treated solids were characterised to determine the total moisture [42], total lignin and polysaccharide contents [27]. Acid-insoluble lignin was determined gravimetrically, while acid-soluble lignin was established using UV spectrophotometry. The content of glucan and hemicelluloses (xylan, arabinan, and acetyl groups) was determined using high performance liquid chromatography (HPLC) equipment. Furthermore, for native biomasses, total extractives, ash and protein contents were determined according to standard methods, namely: NREL/TP-510-42619 [43], NREL/TP-510-42622 [44] and ISO 8968-1:2014 [45], respectively.

The composition of both biomasses is presented in Table 1.

### 3.3. Biomass Processing

#### 3.3.1. Pre-Treatment of Biomass with [emim][HSO_4_]

All reactions were performed with a 10 wt.% of dry biomass in the reaction mixture. For this purpose, 0.5 g of dry biomass and 4.5 g of aqueous IL solution with different [emim][HSO_4_]/H_2_O ratios, were placed into a 15 mL glass vial (Supelco/Sigma-Aldrich, Bellefonte, PA, USA). Next, a vial was placed into the oil bath pre-heated to the desired temperature (140 °C), and reactions were carried out for 90 min, under continuous magnetic stirring. The reaction condition was selected on the basis of previously published work [17]. After reaction, the mixture was cooled to room temperature and approximately 5.0 mL of ultrapure H_2_O was added to precipitate non-hydrolysed fractions. The resulting mixture was filtered under vacuum using 0.45 µm nylon membrane filters. The liquid phase was collected and stored in a freezer for posterior analysis by HPLC, while recovered solid biomass was washed with 100 mL of ultrapure H_2_O (in 10 mL portions) to guarantee removal of IL from the precipitated solid. Next, the obtained solid was placed in the oven at 60 °C for 24 h and afterwards was stored at room temperature for 1 h to analyse the dry mass content. The composition of the solid was analysed as presented in Section 2.2.

#### 3.3.2. Pre-Treatment of Biomass with [emim][OAc]

The pre-treatment was performed according to a method presented in the literature [11]. Reactions were performed at two different temperatures (120 or 140 °C) for 2 h with a 5% (*w*/*w*) biomass/IL ratio in a 15 mL vial. Following the aforementioned procedure presented in the literature, three solid fractions, rich in cellulose, hemicellulose and lignin were obtained. All of them were characterised according to methods presented in Section 2.2.

### 3.4. Enzymatic Hydrolysis of Solids

The digestibility of pre-treated solids obtained with [emim][HSO_4_] or [emim][OAc] was evaluated by enzymatic hydrolysis. The assays were performed using 5% (*w*/*v*) solids (dry weight basis) concentration in 50 mL vials with 5 mL of 0.05 M sodium citrate buffer (pH 5), prepared from citric acid monohydrate and tris-sodium citrate and 100 μL of a 2 wt.% sodium azide solution to prevent undesired growth of microorganisms. Distilled water was added to reach 5.0 mL taking into account the volume of enzyme added last. The enzyme dosage used was 10% (*w*/*w* cellulose) of Celli^®^CTec2 (199.9 FPU·mL^−1^). The enzymatic hydrolyses were performed in a shaking incubator (Optic Ivymen system—Madrid, Spain) at 180 rpm and 50 °C for 72 h. After hydrolysis, enzymes were inactivated by freezing the samples. To measure monosaccharide content, the hydrolysates were filtered under vacuum using nylon filters (pore size of 0.45 μm) and analysed by HPLC. The glucose and xylose yields were calculated considering the glucan and xylan contents and factors of (162/180) and (132/150) for dehydration, respectively.

### 3.5. Chemical Analysis

#### 3.5.1. HPLC Analysis

The liquid phases obtained from the pre-treatment of biomass with [emim][HSO_4_] and enzymatic hydrolyses, as well as from native or pre-treated biomass characterisation were analysed using Agilent 110 series equipment with a Bio-Rad Aminex HPX-87H column (Hercules, CA, USA). Analyses were performed at 65 °C with 5 mmol·L^−1^ H_2_SO_4_ used as the mobile phase at a flow rate of 0.6 mL·min^−1^. The detection was performed using RID (refractive index detector) for monosaccharides (glucose, xylose and arabinose) and acetic acid and DAD (diode array detector) at 280 nm wavelength for furans (furfural and 5-HMF ≡ 5-hydroxymethylfurfural). The quantitative analyses were performed by external calibration using standard solutions.

#### 3.5.2. XRD Measurements

The crystallinity analyses of untreated biomasses and pre-treated materials were performed by X-ray powder diffraction (XRD). For this purpose, a Rigaku Geigerflex D/MAX-III C X-ray powder diffractometer with vertical goniometer, Bragg-Brentano geometry and graphite monochromator was used. The CuKα radiation (λ = 1.5418 Å) at 45 kV and 20 mA was used. The samples were analysed in the range of 2θ from 5° to 50° with increments of 0.02°.

### 3.6. Experimental Uncertainty

Each weighing was made with a standard uncertainty (u) u(m) of 0.1 mg. All pretreatment experiments were performed with a u(T) of 2 °C. All enzymatic hydrolysis experiments were performed with a u(T) of 0.1 °C. All other experimental errors related to measurements depended on the calibration technique used to quantify the concentrations of products. All reactions (pre-treatments and enzymatic hydrolyses) and analyses were performed in duplicate and results are given as mean values with the corresponding standard deviation.

## 4. Conclusions

The present work takes a major step towards providing a comparative framework between two IL-type pre-treatments coupled with enzymatic saccharification, in terms of their performance on converting wheat straw and eucalyptus residues to fermentable sugars. The first strategy relied on the processing of biomass with hydrogen-bond acidic IL. This approach allowed integration of biomass pre-treatment, hydrolysis and conversion of biomass in a single-step process. The acidic character of the [HSO_4_] anion of IL promoted a selective processing of hemicellulose fraction and the resulting products (mainly pentoses and furfural) were kept in a liquid phase. The solids produced were mainly constituted of cellulose and lignin. The second approach with hydrogen-bond basic IL, allowed biomass dissolution and fractionation into cellulose-, hemicellulose- and lignin-rich fractions. In this case, the recalcitrance of the lignocellulosic matrix was overcome by alteration of the strong network of intra- and intermolecular bonds existing in the biomass. Consequently, a loss of native cellulose crystalline I structure was observed, and the cellulose II form was obtained, which was not observed for solids produced by [emim][HSO_4_]. This, in turn allowed a significant improvement in the enzymatic hydrolysis yields to be obtained.

## Figures and Tables

**Table 1:** Wheat straw and eucalyptus residues macromolecular composition.

Components (Dry Weight %)		Wheat Straw	Eucalyptus Residues
Glucan		35.9 ± 0.3	44.1 ± 0.9
Hemicellulose		26.7	19.6
	Xylan	22.1 ± 0.6	15.7 ± 0.2
	Arabinosyl group	2.0 ± 0.7	0.5 ± 0.1
	Acetyl group	2.6 ± 0.9	3.4 ± 0.9
Lignin		16.7	33.8
	Acid-insoluble	15.5 ± 0.4	26.4 ± 0.1
	Acid-soluble	1.2 ± 0.1	7.4 ± 0.1
Ash		11.4 ± 0.1	1.0 ± 0.1
Extractives			
	Water	9.4 ± 1.3	3.3 ± 0.4
	Water (not ash)	5.1 ± 0.5	0.2 ± 0.0
	Ethanol	1.4 ± 0.1	1.5 ± 0.1

## Supplementary Materials

The following are available online: Table S1. Composition of cellulose- and hemicellulose-rich fractions obtained from wheat straw pre-treated with [emim][OAc] at 120 and 140 °C and 2 h; Table S2. Composition of cellulose-rich fractions obtained from eucalyptus pre-treated with [emim][OAc] at 120 and 140 °C and 2 h.
