# Supplementary material for: The Effect of the Chemical Character of Ionic Liquids on Biomass Pre-Treatment and Posterior Enzymatic Hydrolysis
Source: Molecules. 2019 Feb 23;24(4):808. doi: 10.3390/molecules24040808 (PMC6412389; doi:10.3390/molecules24040808)
Supplement: Supplementary file 1 [file molecules-24-00808-s001.pdf]

Electronic Supplementary Information (ESI)

The Effect of the Chemical Character of Ionic Liquids on Biomass Pre-Treatment and Posterior Enzymatic Hydrolysis

Joana R. Bernardo, Francisco M. Gírio and Rafał M. Łukasik *

Unidade de Bioenergia, Laboratório Nacional de Energia e Geologia, I.P., Estrada do Paço do Lumiar 22, 1649-038 Lisboa, Portugal; joana.bernardo@lneg.pt (J.R.B.); francisco.girio@lneg.pt (F.M.G.)

* Correspondence: rafal.lukasik@lneg.pt

Table S1. Composition of cellulose- and hemicellulose-rich fractions obtained from wheat straw pre-treated with [emim][OAc] at 120 and 140 °C and 2 h

	Composition (wt.%)							
T (°C)	Cellulose-rich fraction				Hemicellulose-rich fraction			
	Cellulose	Hemicellulose	Lignin	Others	Cellulose	Hemicellulose	Lignin	Others
120	57.6±0.3	17.1±3.4	17.2±1.0	8.1±3.4	10.8±0.5	61.9±1.5	12.7±0.7	14.7±0.7
140	71.4±0.6	10.3±0.6	10.3±1.8	7.9±0.8	13.1±0.7	67.8±1.6	10.2±0.5	9.0±0.4

Table S2. Composition of cellulose-rich fractions obtained from eucalyptus pre-treated with [emim][OAc] at 120 and 140 °C and 2 h.

	Composition (wt.%)							
T (°C)	Cellulose-rich fraction				Hemicellulose-rich fraction			
	Cellulose	Hemicellulose	Lignin	Others	Cellulose	Hemicellulose	Lignin	Others
120	62.4±0.5	13.9±4.3	23.1±0.5	0.7±0.1	nd	nd	nd	nd
140	62.7±0.8	14.4±0.5	21.9±3.2	1.0±0.6	nd	nd	nd	nd

nd – not determined
